# Factors influencing access of pregnant women and their infants to their local healthcare system: a prospective, multi-centre, observational study

**DOI:** 10.1186/s12884-017-1655-3

**Published:** 2018-01-15

**Authors:** Shabir A. Madhi, Luis M. Rivera, Xavier Sáez-Llorens, Clara Menéndez, Nazira Carrim-Ganey, Mark F. Cotton, Darren Katzman, Mariëtha M. Luttig, Rosalba Candelario, Sherryl Baker, Mahua Roychoudhury

**Affiliations:** 1 Medical Research Council: Respiratory and Meningeal Pathogens Research Unit, University of the Witwatersrand, Johannesburg, South Africa; 2 Department of Science and Technology/National Research Foundation: Vaccine Preventable Diseases, University of the Witwatersrand, Johannesburg, South Africa; 3 National Institute for Communicable Diseases: a division of National Health Laboratory Service, Centre for Vaccines and Immunology, Johannesburg, South Africa; 4 Hospital Universitario Maternidad Nuestra Señora De La Altagracia Perinatology Department, Gazcue, Santo Domingo Dominican Republic; 5 Hospital del Niño “Dr. José Renán Esquivel”, Infectious Disease Department, Panama City, Panama; 6 National System of Investigators (SNI), National Research of the National Secretariat for Science, Technology and Innovation of Panama (SENACYT), Panama City, Panama; 7 Barcelona Institute of Global Health (ISGlobal), Hospital Clinic of Barcelona, Universitat de Barcelona, Barcelona, Spain; 8 Manhiça Health Research Center (CISM), Manhiça, Mozambique; 9 Setshaba Research Centre, Soshanguve, South Africa; 10 Department of Paediatrics and Child Health, Stellenbosch University and Tygerberg Children’s Hospital, Cape Town, South Africa; 11 GSK, Bryanston, South Africa; 12 GSK (formerly employee of Novartis Vaccines Division), Cambridge, MA USA

**Keywords:** Developing countries, Group B *Streptococcus*, Healthcare system, Immunisation, Infants, Pregnant women

## Abstract

**Background:**

The successful implementation of maternal vaccination relies on results of clinical trials, considering the prenatal and postnatal attendance at selected healthcare institutions. This study evaluated factors influencing maternal/infant access to healthcare facilities to identify potential barriers to participation in future clinical trials on maternal vaccination.

**Methods:**

In this prospective, multi-centre, observational study, pregnant women (*N* = 3243) were enrolled at ten sites across Panama, the Dominican Republic, South Africa, and Mozambique between 2012 and 2014. They completed questionnaires at enrolment, delivery, and infant follow-up (90 days post-partum) visits, including questions on transportation, phone accessibility, alternative childcare, gestational age at enrolment, delivery location, and health status of their infant. Logistic regression was used to identify factors significantly associated with return to study site for delivery or infant follow-up visits.

**Results:**

Among 3229 enrolled women with delivery information, 63.6% (range across sites: 25.3–91.5%) returned to study site for delivery. Older women and those at later gestational age at enrolment were more likely to deliver at the study site. While heterogeneities were observed at site level, shorter travel time at delivery and increased transportation costs at enrolment were associated with increased likelihood of women returning to study site for delivery. Among 3145 women with live-born infants, 3077 (95.3%) provided 90-day follow-up information; of these, 68.9% (range across sites: 25.6–98.9%) returned to study site for follow-up visits. Women with other children and with lower transportation costs at delivery were more likely to return to study site for follow-up visits. Among 666 infants reported sick, 94.3% were taken to a healthcare facility, with only 41.9% (range across sites: 4.9–77.3%) to the study site.

**Conclusion:**

Although high retention was observed from enrolment through 90 days after delivery, post-partum surveillance should be broadened beyond the study sites and additional follow-up visits should be planned within the neonatal period. The factors influencing maternal/infant access to healthcare facilities and the issues identified in this study should be taken into consideration in planning future clinical studies on maternal immunisation in low- and middle-income countries.

**Trial registration:**

The study was registered at ClinicalTrial.gov (NCT01734434) on November 22, 2012.

**Electronic supplementary material:**

The online version of this article (10.1186/s12884-017-1655-3) contains supplementary material, which is available to authorized users.

## Background

Between 1990 and 2015, the global under-five annual mortality rate has declined by 53%, from 91 to 43 deaths per 1000 live births [1]. Moreover, an acceleration in the improvement of child survival has been observed during this period, with annual rates of reduction in mortality increasing from 1.8% in 1990–2000 to 3.9% in 2000–2015 [1]. The decline in mortality has been slower in neonates than in post-neonatal under-five children (47% versus 58% globally), with neonatal mortality rates that fell from 36 to 19 deaths per 1000 live births between 1990 and 2015. Almost 45% of the 5.9 million deaths reported worldwide among children younger than 5 years in 2015 occurred in neonates, and the neonatal period remains the most vulnerable time for a child’s survival [1–4]. The vast majority of newborn deaths and almost all maternal deaths (99%) take place in developing countries [3, 5]. As lack of access to quality healthcare at critical time points prior to, during, and after delivery may influence mortality rates [4, 5], identifying barriers to healthcare utilisation could reduce infant and maternal deaths.

Immunisation of pregnant women can protect neonates from diseases which may present within the first days of life: tetanus vaccination is recommended by the World Health Organisation [6], some countries have introduced routine seasonal influenza vaccination [7], and others recommend primary or booster vaccination against pertussis [8, 9]. Maternal vaccination against other diseases, such as Group B *Streptococcus* (GBS), which can kill infants in the first hours of life, is currently under investigation [10, 11]. GBS is a major cause of neonatal sepsis and meningitis, and is classified as early-onset disease when presenting between 0 to 6 days of age and late-onset disease when presenting between 7 to 89 days of age [12]. In previous clinical trials, a single dose of an investigational GBS vaccine was administered to women at 24–35 weeks of gestation, but the optimal vaccination schedule has not been determined [10, 11].

The successful implementation of maternal vaccination will rely on results of clinical trials, taking into account the prenatal and postnatal attendance at the selected healthcare institutions serving as study sites. This study identified factors influencing whether pregnant women would enrol in clinical trials and return to study site for delivery, infant follow-up visits, or medical assistance in case of infant disease. The aim was to identify potential barriers to participation in future large-scale efficacy trials of investigational GBS vaccines administered during pregnancy.

## Methods

This prospective, multi-centre, observational study was conducted between November 2012 and January 2015 in potential sites for a phase 3 maternal immunisation clinical efficacy study in which pregnant women from countries with reported high incidence of GBS disease in infants could be enrolled. Although 13 sites originally qualified for participation, three sites, one each in Malawi, South Africa and Panama, did not recruit any participants. The study was therefore conducted at ten sites: three in Panama, two in the Dominican Republic, four in South Africa, and one in Mozambique (Table 1). The study sites were urban, except the semirural site in Mozambique.

**Table 1:** Demographics and baseline characteristics of women included in the full analysis set

	Panama			Dominican Republic		South Africa				Mozambique
	PA_1	PA_2	PA_3	DR_1	DR_2	SA_1	SA_2	SA_3	SA_4	MO_1
	*N* = 263	*N* = 63	*N* = 174	*N* = 276	*N* = 224	*N* = 500	*N* = 499	*N* = 500	*N* = 490	*N* = 251
Age (years)										
Mean ± SD	24.3 ± 5.2	28.8 ± 6.0	25.0 ± 5.6	24.1 ± 5.1	25.0 ± 5.4	29.0 ± 6.4	25.8 ± 5.9	27.4 ± 5.9	26.0 ± 5.4	25.0 ± 6.3
< 18, n (%)	0 (0)	0 (0)	1 (0.6)	1 (0.4)	2 (0.9)	1 (0.2)	0 (0)	1 (0.2)	0 (0)	0 (0)
18–22, n (%)	113 (43.0)	10 (15.9)	71 (40.8)	132 (47.8)	92 (41.1)	92 (18.4)	182 (36.5)	125 (25.0)	150 (30.6)	111 (44.2)
23–27, n (%)	94 (35.7)	20 (31.7)	50 (28.7)	74 (26.8)	67 (29.9)	134 (26.8)	147 (29.5)	147 (29.4)	164 (33.5)	55 (21.9)
28–32, n (%)	32 (12.2)	13 (20.6)	32 (18.4)	43 (15.6)	40 (17.9)	124 (24.8)	93 (18.6)	126 (25.2)	112 (22.9)	47 (18.7)
33–37, n (%)	19 (7.2)	16 (25.4)	15 (8.6)	25 (9.1)	15 (6.7)	97 (19.4)	50 (10.0)	66 (13.2)	47 (9.6)	28 (11.2)
≥ 38, n (%)	5 (1.9)	4 (6.3)	5 (2.9)	1 (0.4)	8 (3.6)	52 (10.4)	27 (5.4)	35 (7.0)	17 (3.5)	10 (4.0)
Gestational age (weeks)										
Mean ± SD	28.7 ± 3.7	32.7 ± 4.0	29.6 ± 4.4	33.5 ± 4.8	31.1 ± 4.4	32.7 ± 4.6	30.9 ± 2.0	30.7 ± 1.9	30.8 ± 1.8	30.5 ± 2.3
Gestational age calculation method										
Fundal height, n (%)	0 (0)	0 (0)	1 (0.6)	12 (4.3)	1 (0.4)	90 (18.0)	100 (20.0)	263 (52.6)	140 (28.6)	249 (99.2)
Last menstrual period, n (%)	201 (76.4)	36 (57.1)	113 (64.9)	107 (38.8)	139 (62.1)	220 (44.0)	346 (69.3)	181 (36.2)	32 (6.5)	2 (0.8)
Ultrasound, n (%)	62 (23.6)	27 (42.9)	56 (32.2)	156 (56.5)	84 (37.5)	190 (38.0)	53 (10.6)	56 (11.2)	318 (64.9)	0 (0)
Other: Last menstrual period + ultrasound, n (%)	0 (0)	0 (0)	4 (2.3)	0 (0)	0 (0)	0 (0)	0 (0)	0 (0)	0 (0)	0 (0)
Women having other children under care										
n (%)	151 (57.4)	42 (66.7)	92 (52.9)	183 (66.3)	132 (58.9)	304 (60.8)	286 (57.3)	278 (55.6)	217 (44.3)	182 (72.5)
Women caring for other children with alternative childcare provider available										
n (%)	147 (97.4)	37 (88.1)	79 (85.9)	162 (88.5)	124 (93.9)	304 (100)	284 (99.3)	277 (99.6)	215 (99.1)	179 (98.4)

*N* number of women per group, *n (%)* number (percentage) of women in each category, *SD* standard deviation

The study was conducted in accordance with the principles of Good Clinical Practice and the Declaration of Helsinki. The study protocol and related documents were approved by the institutional review boards or ethics committees in each study country: Comite de Docencia e Investigación, Hospital Santo Tomás, Panama; Consejo Nacional de Bioetica en Salud, the Dominican Republic; University of the Witwatersrand Human Research Ethics Committee (Medical), South Africa; Health Research Ethics Committee Research Development and Support Division, Stellenbosh University, South Africa; and National Bioethics Health Committee, Mozambique. Written informed consent was obtained from all women, or their parent/legal representative if minors, prior to enrolment in the study. Informed assent was also obtained from women who were classified as minors. The study was registered at www.clinicaltrials.gov (NCT01734434).

Women who sought antenatal care at the study site were eligible for participation if they were at ≥24 weeks (Panama, the Dominican Republic, and site SA_1 in South Africa) or 28–34 weeks (sites SA_2, SA_3, and SA_4 in South Africa and the site from Mozambique) of gestation at enrolment, and if they provided written informed consent. Following a protocol amendment, the gestational age eligibility window was modified to be consistent with recommendations from the GBS scientific committee specifying that the investigational GBS vaccine should be administered in pregnancy between 28 and 34 weeks of gestation. The new criterion for gestational age was only implemented at three sites from South Africa and the site in Mozambique (SA_2, SA_3, SA_4, and MO_1) because the others had already completed enrolment or were nearing completion at the time of the protocol amendment.

Data were collected from women at each of the three study visits (enrolment, delivery, and infant follow-up visit) by means of questionnaires (Additional file 1). The follow-up visit occurred when the infants reached 90 days of age, which is the upper age limit used to define late-onset GBS infection [12]. The questions were intended to identify the barriers that may hinder access to healthcare or participation in a future clinical trial for pregnant women in each of the settings. Questions asked at all three visits were: duration, cost and type of transportation to the study site, access to a telephone, and availability of care for other children. Specific questions at the enrolment visit (prenatal) were gestational age and its method of estimation, estimated delivery date, and details of the clinic for the woman’s well-child visit (immunisation). At the delivery visit, the time and place of delivery, labour onset, pregnancy outcome, health status of infant, and day of discharge were recorded. Women not delivering at the study site were asked to provide this information telephonically or by completing the non-study site delivery questionnaire. If needed, a home visit was undertaken as per local practice. Additional time points for data collection were the well-child visit (within 21 days after the estimated delivery date) or the 90-day follow-up visit. At the infant follow-up visit (90 days after delivery), the following information was recorded: the health status of the infant, the well-child visit attendance, and whether the infant was sick during the first 90 days of life (in which case further information was recorded on whether medical care was sought or not, where medical care was sought, or the reasons for not seeking medical care). Education about signs and symptoms of potential sepsis in infants was provided to nearly all women (97.3%) at enrolment and at delivery. Women were counselled to seek urgent medical care if their infant developed any symptom of sepsis. Potential GBS infections could be detected if enrolled women identified these specific sepsis symptoms in their child. Investigators recorded all study data on electronic case report forms stored in a secure electronic data capture system for validation.

The study sample size was driven by the enrolment capacity of the sites. Summary statistics were calculated for all recorded questionnaire data, and were presented as frequencies and percentages. Means and standard deviations were presented for continuous variables (e.g. age, gestational age). Analyses were performed on overall data, by region (Latin America or Africa), by country, and by study site. Statistical testing was used only for descriptive purposes, and no multiplicity adjustments were performed.

Stepwise logistic regressions were performed on all enrolled women and on those who provided data after enrolment. Formula and details on odds ratios (ORs) calculation are given in Supplementary Methods (Additional file 2).

To evaluate factors that might influence whether pregnant women would return to study site for delivery, pregnant women were categorised into two groups: those who returned to a study site to deliver and those who did not. Pregnant women whose delivery status was unknown were grouped with those who delivered at a non-study site. To explore the seeking of medical attention, only women with a sick newborn or infant were included for the first occurrence of illness. The predictor variables included in the logistic regressions were: country of study site; age of pregnant woman; gestational age of pregnancy at enrolment; mode, duration, and cost of transportation to the study site; and alternative childcare (no children, children but with no alternative childcare, children with alternative childcare). The stepwise method was used with *p*-values to enter factors set at 0.05 and *p*-values to remove factors set at 0.06. All 95% confidence intervals (CIs) were calculated using the Clopper-Pearson method. All statistical analyses were performed using Statistical Analysis System version 9.1 or a later version.

## Results

### Study participants

**Fig. 1:**
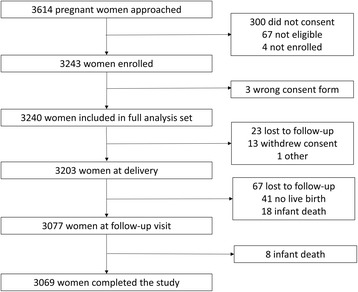
Participant flow diagram

In total, 3614 women were approached for enrolment. Of these, 300 (8.3%) women did not give consent, 67 (1.9%) were not eligible for enrolment, and 4 (0.1%) were not enrolled for other reasons (Fig. 1). Reasons for not participating in the study varied by site; overall, gestational age outside eligibility window was the most common reason. Other reasons included language difficulties, relocation or living outside the study area, family reasons, and lack of interest in participating. Of the 3243 enrolled women, 3069 (94.6%) completed the study. Reasons for premature withdrawal were loss to follow-up (*n* = 90), no live birth (*n* = 41), infant death (*n* = 26), withdrew consent (*n* = 13), signed wrong version of consent form (*n* = 3), and other (*n* = 1). Of note, the proportion of women who withdrew because they had no live birth was higher in Africa (38 [1.7%; range across sites: 1.0–2.4%]) than in Latin America (3 [0.3%; range across sites: 0.0–0.6%]), and among the 26 women prematurely withdrawn from the study for infant death, 4 (0.4%; range across sites: 0.0–0.7%) were in Latin America and 22 (1.0%; range across sites: 0.2–2.0%) in Africa.

The mean age and gestational age of enrolled women were similar across sites (Table 1). The method to determine gestational age varied by site and/or country. Fundal height was predominant in Mozambique (99.2% of women) and at one site in South Africa (site SA_3: 52.6%), compared with last menstrual period (LMP) or ultrasound at the other sites. An alternative childcare provider was available at enrolment for 96.8% (range across sites: 85.9–100%) of women caring for other children.

Most women were contactable by phone at all sites for each study visit (range across sites: 75.7–100%). Of those contactable, 99.4–100% of women in Mozambique were contactable by personal mobile phone for the three visits. Personal mobile phone use was also high in South Africa (range across sites: 96.9–99.8%), but slightly lower in the Dominican Republic and Panama (75.8–84.0% and 85.2–96.6%, respectively). No women in Mozambique were contactable by home phone. Home phone access was also low in South Africa (0.2–5.2%), but was higher in the Dominican Republic (18.2–27.8%) and Panama (29.3–44.4%).

### Factors influencing delivery at study site

Among the 3229 enrolled women with delivery information, 2053 (63.6%) delivered at the study site, and 1115 (34.5%) delivered at a non-study healthcare facility (Table 2). There were large differences in percentages of women returning to the study site for delivery, ranging from 25.3–91.5% across sites. Reasons for a non-study site delivery were multiple (categorised as “other”) for most women (79.9%; range across sites: 5.0–95.3%), but the most common reason given was “labour too fast” (10.0%; range across sites: 2.5–70.0%) (Table 2).

**Table 2:** Study site and non-study site deliveries for women included in full analysis set with available delivery information

	Panama			Dominican Republic		South Africa				Mozambique
	PA_1	PA_2	PA_3	DR_1	DR_2	SA_1	SA_2	SA_3	SA_4	MO_1
	*N* = 263	*N* = 63	*N* = 174	*N* = 272	*N* = 224	*N* = 497	*N* = 499	*N* = 497	*N* = 489	*N* = 251
Delivery location										
Study site, n (%)	203 (77.2)	43 (68.3)	126 (72.4)	249 (91.5)	91 (40.6)	422 (84.9)	126 (25.3)	414 (83.3)	161 (32.9)	218 (86.9)
Non-study healthcare facility, n (%)	57 (21.7)	18 (28.6)	48 (27.6)	21 (7.7)	132 (58.9)	68 (13.7)	357 (71.5)	79 (15.9)	315 (64.4)	20 (8.0)
Home, n (%)	0 (0)	2 (3.2)	0 (0)	0 (0)	0 (0)	3 (0.6)	7 (1.4)	2 (0.4)	3 (0.6)	7 (2.8)
Other, n (%)	0 (0)	0 (0)	0 (0)	0 (0)	0 (0)	2 (0.4)	5 (1.0)	1 (0.2)	2 (0.4)	1 (0.4)
Unknown, n (%)	3 (1.1)	0 (0)	0 (0)	2 (0.7)	1 (0.5)	2 (0.4)	4 (0.8)	1 (0.2)	8 (1.6)	5 (2.0)
Reason for non-study site delivery										
Labour too fast, n (%)	28 (49.1)	14 (70.0)	8 (16.7)	7 (33.3)	5 (3.8)	15 (20.6)	12 (3.3)	8 (9.6)	8 (2.5)	10 (35.7)
Did not want to deliver at site, n (%)	5 (8.8)	1 (5.0)	7 (14.6)	1 (4.8)	7 (5.3)	10 (13.7)	5 (1.4)	9 (10.8)	1 (0.3)	0 (0)
Family did not allow, n (%)	1 (1.8)	0 (0)	1 (2.1)	0 (0)	0 (0)	5 (6.9)	4 (1.1)	15 (18.1)	8 (2.5)	0 (0)
No direct transportation to site, n (%)	0 (0)	2 (10.0)	19 (39.6)	0 (0)	0 (0)	0 (0)	1 (0.3)	2 (2.4)	0 (0)	3 (10.7)
No money for transportation, n (%)	0 (0)	2 (10.0)	10 (20.8)	0 (0)	0 (0)	2 (2.7)	1 (0.3)	0 (0)	0 (0)	0 (0)
No childcare available for other children, n (%)	0 (0)	0 (0)	0 (0)	0 (0)	1 (0.8)	0 (0)	1 (0.3)	0 (0)	0 (0)	1 (3.6)
Other^a^, n (%)	25 (43.9)	1 (5.0)	3 (6.3)	14 (66.7)	120 (90.9)	41 (56.2)	349 (94.6)	48 (57.8)	305 (95.3)	14 (50.0)

*N* number of women per group, *n (%)* number (percentage) of women in each category

^a^Other: includes another hospital or healthcare centre was closer, women had a healthcare insurance coverage or a relative working at another healthcare facility, no free beds at study site, pregnancy complications/emergencies, away from area at time of labour

Both mean age and gestational age were significant factors influencing whether a woman delivered at the study site, with older women and women at later gestational age at enrolment being more likely to deliver at the study site. With every year increase in the woman’s age and with every week increase in the gestational age, the participant was more likely to deliver at the study site (ORs = 1.02 [95% CI: 1.01, 1.04] and 1.01 [95% CI: 1.01, 1.01], respectively) (Table 3).

**Table 3:** Predictor variables included in the logistic regression model for study site delivery and follow-up visit

	Parameter estimate	Odds ratio (95% CI)	*p*-value
Factors influencing delivery at the study site (full analysis set)			
Country of study site			
Dominican Republic vs South Africa	−0.61	1.15 (0.87, 1.52)	<0.0001
Mozambique vs South Africa	1.19	6.96 (4.14, 11.69)	<0.0001
Panama vs South Africa	0.17	2.50 (1.93, 3.23)	0.1391
Age of the pregnant women	0.02	1.02 (1.01, 1.04)	0.0008
Gestational age	0.01	1.01 (1.01, 1.01)	<0.0001
Mode of transportation			
Minibus/Taxi - Visit 1 (Yes vs No)	0.11	1.26 (1.05, 1.50)	0.0123
Public transportation - Visit 2 (Yes vs No)	0.16	1.39 (1.07, 1.80)	0.0124
Walk - Visit 2 (Yes vs No)	0.47	2.56 (1.62, 4.04)	<0.0001
Ambulance - Visit 2 (Yes vs No)	−0.73	0.23 (0.18, 0.30)	<0.0001
Duration of transportation to study site (Visit 1)			
< 15 min vs 15–29 min	−0.35	0.73 (0.58, 0.92)	0.0131
30–44 min vs 15–29 min	−0.06	0.98 (0.79, 1.21)	0.6716
45–59 min vs 15–29 min	0.33	1.43 (1.03, 1.99)	0.0477
60–89 min vs 15–29 min	0.19	1.26 (0.88, 1.80)	0.2515
90–120 min vs 15–29 min	−0.11	0.93 (0.50, 1.73)	0.6957
> 120 min vs 15–29 min	0.03	1.06 (0.31, 3.66)	0.9592
Duration of transportation to study site (Visit 2)			
< 15 min vs 15–29 min	0.70	1.52 (1.15, 2.02)	<0.0001
30–44 min vs 15–29 min	−0.42	0.50 (0.41, 0.61)	0.0007
45–59 min vs 15–29 min	−0.09	0.69 (0.52, 0.93)	0.5444
60–89 min vs 15–29 min	0.04	0.79 (0.56, 1.12)	0.8159
90–120 min vs 15–29 min	0.23	0.96 (0.50, 1.85)	0.4259
> 120 min vs 15–29 min	−0.74	0.36 (0.11, 1.18)	0.1507
Cost of transportation to study site (Visit 1)	0.08	1.09 (1.02, 1.16)	0.0133
Factors influencing return to the study site for follow-up visit (full analysis set with live-born infants)			
Country of study			
Dominican Republic vs South Africa	2.40	19.20 (10.16, 36.27)	<0.0001
Mozambique vs South Africa	−1.32	0.46 (0.32, 0.67)	<0.0001
Panama vs South Africa	−0.52	1.03 (0.82, 1.29)	<0.0001
Mode of transportation to study site			
Walk - Visit 2 (Yes vs No)	0.31	1.85 (1.21, 2.81)	0.0042
Cost of transportation to study site at Visit 2	−0.01	0.99 (0.97, 1.00)	0.0257
Alternative Childcare at Visit 1			
Alternative care available vs No other children	0.21	1.35 (1.14, 1.60)	0.1115

*CI* confidence intervals, *Visit 1* enrolment, *Visit 2* delivery

Mode of transportation at enrolment or delivery was similar (data shown for delivery) for all sites within each country, but varied between countries (Table 4). At the delivery visit, the most common modes of transportation in Panama and South Africa were minibus/taxi (43.2% [range across sites: 31.2–62.1%] and 34.3% [19.0–44.8%], respectively) and private car (35.6% [26.4–41.1%] and 38.5% [34.2–44.9%], respectively), while public transport (40.4% [32.1–47.1%]) and minibus/taxi (35.6% [22.5–51.8%]) were most common in the Dominican Republic and walking (53.4%) in Mozambique. When compared to other modes of transportation and holding all other variables constant in the model, women who used minibus/taxi (OR = 1.26 [95% CI: 1.05, 1.50]) at enrolment and those who used public transportation (OR = 1.39 [95% CI: 1.07, 1.80]) or walking (OR = 2.56 [95% CI: 1.62, 4.04]) at delivery were more likely to deliver at the study site, while those reaching the study site by ambulance for delivery (OR = 0.23 [95% CI: 0.18, 0.30]) were less likely to deliver there (Table 3).

**Table 4:** Transportation to site/healthcare facilities at delivery for women included in the full analysis set

	Panama			Dominican Republic		South Africa				Mozambique
	PA_1	PA_2	PA_3	DR_1	DR_2	SA_1	SA_2	SA_3	SA_4	MO_1
	*N* = 263	*N* = 63	*N* = 174	*N* = 276	*N* = 224	*N* = 500	*N* = 499	*N* = 500	*N* = 490	*N* = 251
Mode of transportation^a^										
Ambulance, n (%)	13 (4.9)	10 (15.9)	3 (1.7)	0 (0)	0 (0)	69 (13.8)	35 (7.0)	32 (6.4)	174 (35.5)	11 (4.4)
Minibus/taxi, n (%)	82 (31.2)	26 (41.3)	108 (62.1)	62 (22.5)	116 (51.8)	224 (44.8)	173 (34.7)	192 (38.4)	93 (19.0)	5 (2.0)
Private car, n (%)	108 (41.1)	24 (38.1)	46 (26.4)	64 (23.2)	26 (11.6)	189 (37.8)	224 (44.9)	171 (34.2)	182 (37.1)	44 (17.5)
Public transportation, n (%)	60 (22.8)	4 (6.3)	19 (10.9)	130 (47.1)	72 (32.1)	1 (0.2)	44 (8.8)	75 (15.0)	5 (1.0)	41 (16.3)
Walk, n (%)	8 (3.0)	0 (0)	0 (0)	41 (14.9)	12 (5.4)	11 (2.2)	16 (3.2)	10 (2.0)	16 (3.3)	134 (53.4)
Other, n (%)	0 (0)	0 (0)	0 (0)	24 (8.7)	5 (2.2)	0 (0)	1 (0.2)	7 (1.4)	0 (0)	4 (1.6)
Duration of travel										
< 15 min, n (%)	13 (4.9)	5 (7.9)	39 (22.4)	17 (6.2)	26 (11.6)	76 (15.2)	41 (8.2)	73 (14.6)	46 (9.4)	7 (2.8)
15–29 min, n (%)	85 (32.3)	23 (36.5)	54 (31.0)	59 (21.4)	83 (37.1)	209 (41.8)	219 (43.9)	295 (59.0)	199 (40.6)	20 (8.0)
30–44 min, n (%)	61 (23.2)	11 (17.5)	39 (22.4)	71 (25.7)	76 (33.9)	133 (26.6)	170 (34.1)	80 (16.0)	163 (33.3)	85 (33.9)
45–59 min, n (%)	23 (8.7)	7 (11.1)	16 (9.2)	49 (17.8)	19 (8.5)	65 (13.0)	35 (7.0)	27 (5.4)	38 (7.8)	85 (33.9)
60–89 min, n (%)	61 (23.2)	14 (22.2)	16 (9.2)	56 (20.3)	14 (6.3)	8 (1.6)	20 (4.0)	8 (1.6)	17 (3.5)	30 (12.0)
90–120 min, n (%)	11 (4.2)	1 (1.6)	6 (3.4)	17 (6.2)	4 (1.8)	3 (0.6)	7 (1.4)	3 (0.6)	2 (0.4)	9 (3.6)
> 120 min, n (%)	4 (1.5)	0 (0)	3 (1.7)	1 (0.4)	0 (0)	0 (0)	0 (0)	1 (0.2)	2 (0.4)	3 (1.2)
Cost ($)										
mean ± SD	5.4 ± 6.5	5.9 ± 6.0	5.2 ± 4.5	3.6 ± 3.8	4.8 ± 3.1	5.1 ± 6.7	3.8 ± 6.6	5.0 ± 6.8	4.9 ± 7.0	0.3 ± 1.0

*N* number of women per group, *n (%)* number (percentage) of women in each category, *SD* standard deviation

^a^The women participating in the study were allowed to indicate more than 1 way of transportation, the most frequent combination was “public transportation & walk”

Within a country, travel times to the study site were similar (Table 4). In Mozambique, most women travelled 30–59 min (67.8%) to reach the study site for delivery. The majority in the three other countries travelled less than 45 min (66.4% in the Dominican Republic, 66.0% in Panama, and 85.7% in South Africa). A short duration of transportation at delivery was positively associated with women returning to the study site for delivery. When comparing women with 15–29 min of transportation to reach the study site for delivery, those with less than 15 min were more likely (OR = 1.52 [95% CI: 1.15, 2.02]) and those with 30–44 min less likely (OR = 0.50 [95% CI: 0.41, 0.61]) to deliver at the study site (Table 3).

The mean direct cost of transportation at delivery was similar at the sites in Panama, the Dominican Republic, and South Africa ($3.6–$5.9), but much lower in Mozambique ($0.3). With every unit increase in cost of transportation (in United States dollar [USD]) at enrolment, the women were 1.09 (95% CI: 1.02, 1.16) times more likely to deliver at the study site (Table 3).

### Outcome at the 90-day infant follow-up visit

**Fig. 2:**
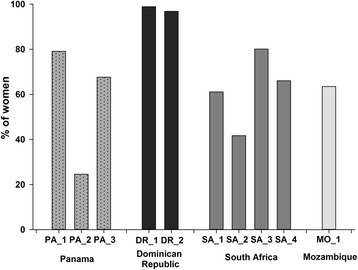
Percentage of women returning to the study site for infant follow-up visit, 90 days post-delivery

In total, 97.8% of women with live-born infants (3077/3145) provided 90-day infant follow-up information, but only 2121 of them (68.9%) returned to the study site for this visit. The overall percentage of women returning to the study site was similar in Mozambique, Panama, and South Africa (62.2–68.2%), but much higher in the Dominican Republic (98.0%), with significant inter-site differences within countries (Fig. 2). At the 90-day infant follow-up visit, information was available for 3106 infants, of whom 3089 (99.5%) attended at least one well-child visit at the study site or another healthcare facility.

Women who walked to the site for delivery were significantly more likely to return to the study site for the infant follow-up visit (OR = 1.85 [95% CI: 1.21, 2.81]). Cost of transportation calculated at delivery was also a significant factor influencing whether women returned to the study site for the infant follow-up visit. With every unit increase in cost of transportation (in USD) at delivery, the women were 0.99 (95% CI: 0.97, 1.00) times less likely to return to the study site for their follow-up visit (Table 3).

Alternative childcare availability was very high (96.8%; range across sites: 85.9–100%) (Table 1), and women with alternative childcare provider availability at enrolment, representing women with children, were more likely to return to the study site for follow-up than those without other children (OR = 1.35 [95% CI: 1.14, 1.60) (Table 3).

### Sick child reporting and medical care

Of the 3199 live born infants, 218 (6.8%; range across sites: 2.0–19.3%) newborns were reported sick before discharge. Overall, the highest percentage of newborns reported sick was in Panama (16.3%) and the lowest in Mozambique (2.1%), but this percentage varied across sites within each country. At the 90-day infant follow-up visit, 666/3106 (21.4%; range across sites: 3.7–68.8%) infants were reported sick during the first 90 days of life; this percentage was lower in Africa (11.3%) compared to Latin America (43.3%). Of these 666 infants, 94.3% (range across sites: 82.2–100%) were taken to a healthcare facility, and 41.9% (range across sites: 4.9–77.3%) were taken to the study site (Table 5). The most common reason for the sick infant not being taken to a healthcare facility was that the infant did not appear seriously ill (31/38 infants, 81.6%). Other factors were lack of money and “other” (e.g. infant already hospitalised, infant died before reaching healthcare).

**Table 5:** Medical assistance for infants reported sick within 90 days post-delivery

	Panama			Dominican Republic		South Africa				Mozambique
	PA_1	PA_2	PA_3	DR_1	DR_2	SA_1	SA_2	SA_3	SA_4	MO_1
	*N* = 254	*N* = 61	*N* = 176	*N* = 269	*N* = 221	*N* = 487	*N* = 480	*N* = 485	*N* = 441	*N* = 232
Infant reported sick in first 90 days										
n (%)	127 (50.0)	32 (52.5)	121 (68.8)	104 (38.7)	41 (18.6)	18 (3.7)	41 (8.5)	43 (8.9)	73 (16.6)	66 (28.5)
Sick infant taken to										
Study site, n (%)	58 (45.7)	12 (37.5)	77 (63.6)	27 (26.0)	27 (65.9)	7 (38.9)	2 (4.9)	13 (30.2)	5 (6.9)	51 (77.3)
Infant’s hospital/immunisation clinic, n (%)	2 (1.6)	6 (18.8)	7 (5.8)	10 (9.6)	3 (7.3)	10 (55.6)	13 (31.7)	10 (23.3)	21 (28.8)	13 (19.7)
Other, n (%)	65 (51.2)	14 (43.8)	34 (28.1)	61 (58.7)	8 (19.5)	1 (5.6)	22 (53.7)	15 (34.9)	34 (46.6)	0 (0)
Not taken to healthcare facility, n (%)	2 (1.6)	0 (0)	3 (2.5)	6 (5.8)	3 (7.3)	0 (0)	4 (9.8)	5 (11.6)	13 (17.8)	2 (3.0)
Reasons for not taking sick infant to healthcare facility										
Newborn did not appear seriously ill, n (%)	0 (0)	0 (0)	3 (100)	4 (66.7)	3 (100)	0 (0)	3 (75.0)	4 (80.0)	13 (100)	1 (50.0)
Lack of money, n (%)	1 (50.0)	0 (0)	0 (0)	1 (16.7)	0 (0)	0 (0)	0 (0)	0 (0)	0 (0)	0 (0)
Lack of transportation, n (%)	0 (0)	0 (0)	0 (0)	0 (0)	0 (0)	0 (0)	0 (0)	0 (0)	0 (0)	0 (0)
No alternative childcare available, n (%)	0 (0)	0 (0)	0 (0)	0 (0)	0 (0)	0 (0)	0 (0)	0 (0)	0 (0)	0 (0)
Other, n (%)	1 (50.0)	0 (0)	0 (0)	1 (16.7)	0 (0)	0 (0)	1 (25.0)	1 (20.0)	0 (0)	1 (50.0)

*N* infants with information available at 90 days follow-up, *n (%)* number (percentage) of subjects in each category

## Discussion

This study identified factors influencing whether pregnant women would enrol in clinical trials, and would return to study sites for delivery, infant follow-up visits, or to seek medical assistance for their sick infant. By identifying restrictions to maternal/infant healthcare access in potential clinical sites, this study provided a potential platform for improving compliance and retention for future phase 3 clinical efficacy studies of investigational GBS vaccines. Apart from greater efficiency in clinical trials, there is evidence that ante- and post-partum visits improve maternal and infant health, and increase perinatal infant survival rates by offering opportunities for education and medical interventions. A greater understanding of predictors of pregnant women access to healthcare services may therefore also have programmatic implications for the improvement of healthcare utilisation.

Out of all pregnant women who were approached, 10% were not eligible for the study and could not participate. The reasons for not participating were estimated with the most common being lower than required gestational age. Proportions of ineligible women and reasons for ineligibility will be useful in the operational planning of clinical trials enrolling pregnant women, e.g. women with too low gestational age at screening could potentially participate if they return later during pregnancy.

Women at later gestational age at enrolment were more likely to deliver at the study site. In clinical trials, the gestational age is estimated to identify the appropriate time for maternal vaccination to ensure optimum placental antibody transfer to the infant [13]. Here, methods of estimating gestational age varied among sites, the most common being ultrasound or LMP in Latin America and South Africa, and fundal height in Mozambique. Ultrasound is considered the most accurate method, but is less available in resource-limited settings [13–15]. The LMP method can be universally applied, but relies on self-assessment and reporting, and is potentially unreliable [14]. The fundal height method is routinely used in many resource-limited countries [16].

Older women were also more likely to return to study sites for delivery. This observation is in line with previous studies suggesting that women in their 30s attended antenatal healthcare more frequently than younger or older women, but contrasts with other studies suggesting that age had no effect on antenatal healthcare utilisation [17]. Another finding was that women with other children were more likely to return to study site for follow-up visits. A potential explanation for this unexpected finding could be that multiparous women often return to the site of their previous delivery for antenatal visits and are therefore more likely to return to the same site for delivery and follow-up visits.

Additionally, we identified factors that may hinder access to healthcare facilities for pregnant women and their infants. Mode, duration, and cost of transportation had a significant influence on whether women would return to study site for delivery and follow-up visits. Transportation was previously identified as a barrier for access to obstetric care in low- and middle-income countries, with increasing cost, distance, and travel time reducing the likelihood of returning [17–20]. Surprisingly, we found that increased transportation costs at enrolment, which were lower than transportation costs at delivery, were associated with increased likelihood of women returning to study sites for delivery. This observation suggests that women who can afford to pay for transportation to antenatal visits are more likely to return to the same site for delivery. Another potential explanation could be that women living further from the study site may stay nearby when their due date was coming closer, which would then decrease the transportation costs at delivery. In contrast, lower transportation costs at delivery were associated with an increased likelihood of women returning to the study site for follow-up visits. Our results suggest that transportation cost is a barrier for routine follow-up visits, but not for hospital delivery, which is an important finding to consider when preparing clinical trials on maternal immunisation. In Africa, this finding should be interpreted with caution considering the differences in minimum salary in the respective countries [21].

Over 90% of women sought medical care at the study site or another healthcare facility for their sick infant, suggesting that the probability to detect potential GBS infections was high. However, more widespread efforts are needed to collect detailed follow-up information since only two thirds of live born infants were brought to the study site for the 90-day follow-up visits. Each site from a clinical study should establish surveillance in surrounding facilities where sick infants may be seen.

The study had a number of limitations. Although education about signs and symptoms of sepsis was provided to nearly all women, the study failed to clearly define what “sick” or “illness” meant, allowing thresholds for considering a child as ill that could differ by country. In addition, the incidence and causes of disease in infants were not collected as the majority of sick children were not assessed at the study site. When choosing sites for potential phase 3 trials on investigational maternal GBS vaccines, it is important to understand the neonatal invasive GBS disease incidence in the area; however, these estimates are lacking for many low- and middle-income countries [22]. The low proportion of women younger than 18 years of age (0.2%) was another potential limitation. Indeed, 16% of pregnant women in sub-Saharan Africa are under 19 years [23]; these young women are often at higher risk for GBS due to a lack of sexual health education [24] and a lower level of natural immunity against colonisation. Young pregnant women are an important group to study because they are often underrepresented in clinical trials due to legal or logistical reasons. This study was also limited by the fact that only healthcare sites large enough to conduct phase 3 clinical trials were used. These were in large urban areas, except the semirural site in Mozambique. Therefore, factors influencing whether women would return to healthcare facilities may vary for rural areas and smaller healthcare sites. The absence of data on the following variables was a further limitation of this study: the mode of delivery, the occurrence of complications during delivery, and the quality of the antenatal and birthing experience, which may all impact the likelihood of returning for a follow-up visit. Finally, failure to collect information on the impact of other important factors, such as education, parity, and income, was another limitation [25, 26].

## Conclusions

In this prospective, multi-centre, observational study, older pregnant women and those at later gestational age at enrolment were more likely to deliver at the study site. Women with other children were more likely to return to study site for follow-up visits. While heterogeneities were observed at site level, shorter travel time at delivery and increased transportation costs at enrolment were associated with increased likelihood of women returning to study site for delivery. Lower transportation costs at delivery were associated with increased likelihood of women returning to study site for follow-up visits. Although high retention was observed from enrolment through 90 days after delivery, post-partum surveillance should be broadened beyond the study sites and an additional follow-up visit should be planned within the first month of life, the most vulnerable time for a child’s survival. The factors influencing maternal/infant access to healthcare facilities and the issues identified in this study should be taken into consideration in planning future clinical studies on maternal immunisation in low- and middle-income countries.

## Additional files

Additional files 1: Survey Questionnaires. Survey questionnaires completed at enrolment, delivery (at study site or outside of study site) and infant 90-day follow-up visit. (PDF 57 kb)

Additional files 2: Supplementary Methods. Formula and details on odds ratios calculation. (PDF 85 kb)

## Abbreviations

CI: Confidence interval
GBS: Group B *Streptococcus*
LMP: Last menstrual period
OR: Odds ratio
USD: United States dollar
