# Supplementary material for: Factors influencing access of pregnant women and their infants to their local healthcare system: a prospective, multi-centre, observational study
Source: BMC Pregnancy Childbirth. 2018 Jan 15;18:29. doi: 10.1186/s12884-017-1655-3 (PMC5769295; doi:10.1186/s12884-017-1655-3)
Supplement: Supplementary file 1 — Survey Questionnaires. Survey questionnaires completed at enrolment, delivery (at study site or outside of study site) and infant 90-day follow-up visit. (PDF 57 kb) [file 12884_2017_1655_MOESM1_ESM.pdf]

Additional file 1

Factors influencing access of pregnant women and their infants to their local healthcare system: a prospective, multi-centre, observational study

Shabir A. Madhi et al.

Survey Questionnaires.

Survey questionnaire: Enrolment

Survey questionnaire: Delivery at study site

Survey questionnaire: Delivery outside of study site

Survey questionnaire: Infant 90-day follow-up visit

Survey Questionnaire: Enrolment

1. Date of visit : _ _/_ _ _/_ _ _ _ (dd/mmm/yyyy)

2. What type of transportation did you use to reach the study site? (Include all applicable)
   - ☐ Public transportation
   - ☐ Minibus/taxi
   - ☐ Private car
   - ☐ Walk
   - ☐ Ambulance
   - ☐ Other____________________________

3. How much time did it take to reach the study site?
   - ☐ Less than 15 min
   - ☐ Between 15 and 29 mins
   - ☐ Between 30 and 44 mins
   - ☐ Between 45 and 59 mins
   - ☐ Between 60 and 89 mins
   - ☐ Between 90 and 120 mins
   - ☐ More than 120 mins

4. How much did it cost to reach the study site?
   ____ (Please also indicate the currency)

5. Do you have access to a telephone to which you can be contacted by the study staff?
   - ☐ No
   - ☐ Yes, provide all numbers here.

     Home phone___________________________________
     Personal mobile phone___________________________
     Other____
     (Phone number(s) is(are) only for the register of the site. It will not be included in the eCRF)

6. Do you have other child(ren) under your care at this time?
   - ☐ No
   - ☐ Yes. Is anyone else looking after them while you are in hospital?
     - ☐ No
     - ☐ Yes

7. What is the name of the clinic to which you will bring your newborn for well child and immunization visits? (This information is only for the register of the site. It will not be included in the eCRF)

For site staff:

8. Have you informed the subject about signs and symptoms of potential sepsis in infants?
    - ☐ No
    - ☐ Yes

Survey Questionnaire: Delivery at study site

1. Did the subject deliver at study site?
   - ☐ No (If no, please complete the questionnaire for delivery outside of study site)
   - ☐ Yes (If yes, please complete the following questions)

2. Date of visit: _ _/_ _ _/_ _ _ _ (dd/mmm/yyyy)

3. What type of transportation did you use to reach the study site? (Include all applicable)
   - ☐ Public transportation
   - ☐ Minibus/taxi
   - ☐ Private car
   - ☐ Walk
   - ☐ Ambulance
   - ☐ Other___________________________

4. How much time did it take to reach the study site?
   - ☐ Less than 15 min
   - ☐ Between 15 and 29 mins
   - ☐ Between 30 and 44 mins
   - ☐ Between 45 and 59 mins
   - ☐ Between 60 and 89 mins
   - ☐ Between 90 and 120 mins
   - ☐ More than 120 mins

5. How much money did it cost to reach the study site?
   _______________________________________ (Please also indicate the currency)

6. Do you have access to a telephone to which you can be contacted by the study staff?
   - ☐ No
   - ☐ Yes, provide all numbers here.
     Home phone__________________________________
     Personal mobile phone__________________________
     Other____
     (Phone number(s) is(are) only for the register of the site. They will not be included in the eCRF)

7. Do you have other child(ren) under your care at this time?
   - ☐ No
   - ☐ Yes. Is anyone else looking after them while you are in hospital?
     - ☐ No
     - ☐ Yes

8. Date of labor onset: _ _/_ _ _/_ _ _ _ (dd/mmm/yyyy)

9. Time of labor onset: _ _/_ _ ☐AM ☐PM

10. Date of arrival at site for delivery: _ _/_ _ _/_ _ _ _ (dd/mmm/yyyy)

11. Time of arrival at site for delivery: _ _/_ _ ☐AM ☐PM

12. Date of delivery: _ _/_ _ _/_ _ _ _ (dd/mmm/yyyy)

13. Result of pregnancy: No. of living infants: ☐0 ☐1 ☐2 ☐3 ☐4

14. For infant 1: ☐N/A
    Date of discharge: _ _/_ _ _/_ _ _ _ (dd/mmm/yyyy)
    Time of discharge: _ _:_ _ ☐AM ☐PM
    Health status at discharge: ☐Alive ☐Not alive
    Was the infant sick at any time since birth: ☐No ☐Yes

15. For infant 2: ☐N/A
    Date of discharge: _ _/_ _ _/_ _ _ _ (dd/mmm/yyyy)
    Time of discharge: _ _:_ _ ☐AM ☐PM
    Health status at discharge: ☐Alive ☐Not alive
    Was the infant sick at any time since birth: ☐No ☐Yes

16. For infant 3: ☐N/A
    Date of discharge: _ _/_ _ _/_ _ _ _ (dd/mmm/yyyy)
    Time of discharge: _ _:_ _ ☐AM ☐PM
    Health status at discharge: ☐Alive ☐Not alive
    Was the infant sick at any time since birth: ☐No ☐Yes

17. For infant 4: ☐N/A
    Date of discharge: _ _/_ _ _/_ _ _ _ (dd/mmm/yyyy)
    Time of discharge: _ _:_ _ ☐AM ☐PM
    Health status at discharge: ☐Alive ☐Not alive
    Was the infant sick at any time since birth: ☐No ☐Yes

For site staff:

18. Have you informed the subject about signs and symptoms of potential sepsis in infants?
    - ☐ No
    - ☐ Yes

Survey Questionnaire: Delivery outside of study site

1. Did the subject deliver at a site that is not from the study?
   - ☐ No (If no, please complete the questionnaire for delivery at study site)
   - ☐ Yes (If yes, please complete the following questions)

2. Place of delivery
   - ☐ Non-study healthcare facility. Provide the name of the facility.
     ____
   - ☐ Home
   - ☐ Other____________________________

3. What type of transportation did you use to reach the healthcare facility? (Include all applicable)
   - ☐ N/A (For home delivery)
   - ☐ Public transportation
   - ☐ Minibus/taxi
   - ☐ Private car
   - ☐ Walk
   - ☐ Ambulance
   - ☐ Other____________________________

4. How much time did it take to reach the healthcare facility?
   - ☐ N/A (For home delivery)
   - ☐ Less than 15 min
   - ☐ Between 15 and 29 mins
   - ☐ Between 30 and 44 mins
   - ☐ Between 45 and 59 mins
   - ☐ Between 60 and 89 mins
   - ☐ Between 90 and 120 mins
   - ☐ More than 120 mins

5. How much money did it cost to reach the healthcare facility?
   - ☐ N/A (For home delivery)

   ____ (Please also indicate the currency)

6. Do you have access to a phone to which you can be contacted by the study staff?
   - ☐ No
   - ☐ Yes, provide all numbers here.

     Home phone___________________________________

     Personal mobile phone___________________________

Other____
(Phone number(s) is(are) only for the register of the site. It will not be included in the eCRF)

7. Do you have other child(ren) under your care at this time?
   - ☐ No
   - ☐ Yes. Is anyone else looking after them while you are in hospital?
     - ☐ No
     - ☐ Yes
     - ☐ N/A (For home delivery)

8. Date of onset of labor: _ _/_ _ _/_ _ _ _ (dd/mmm/yyyy)

9. Time of onset of labor: _ _/_ _ ☐AM ☐PM

10. Date of arrival at healthcare facility for delivery: _ _/_ _ _/_ _ _ _ (dd/mmm/yyyy)
    - ☐ N/A (For home delivery)

11. Time of arrival at healthcare facility for delivery: _ _/_ _ ☐AM ☐PM
    - ☐ N/A (For home delivery)

12. Date of delivery: _ _/_ _ _/_ _ _ _ (dd/mmm/yyyy)

13. Result of pregnancy: No. of living infants: ☐0 ☐1 ☐2 ☐3 ☐4

For infant 1: ☐N/A

14. Date of discharge: _ _/_ _ _/_ _ _ _ (dd/mmm/yyyy) N/A (For home delivery)
15. Time of discharge: _ _/_ _ ☐AM ☐PM N/A (For home delivery)
16. Health status at discharge: ☐Alive ☐Not alive N/A (For home delivery)
17. Health status at the time of the contact: ☐Alive ☐Not alive
18. If not alive, was infant sick at any time since birth?
    ☐No ☐Yes
19. If yes, was the sick infant brought to healthcare facility for medical care? ☐No ☐Yes
20. If no, what is(are) the reason(s) for not bringing the sick infant to a healthcare facility?
    - ☐ No alternative child care
    - ☐ Lack of transportation
    - ☐ Lack of money
    - ☐ The infant did not appear to be seriously ill
    - ☐ Other__________________________

For infant 2: ☐N/A

21. Date of discharge: _ _/_ _ _/_ _ _ _ (dd/mmm/yyyy) N/A (For home delivery)
22. Time of discharge: _ _/_ _ ☐AM ☐PM N/A (For home delivery)
23. Health status at discharge: ☐Alive ☐Not alive N/A (For home delivery)

24. Health status at the time of the contact: □Alive □Not alive

25. If not alive, was infant sick at any time since birth?

□No □Yes

26. If yes, was the sick infant brought to healthcare facility for medical care? □No □Yes

27. If no, what is(are) the reason(s) for not bringing the sick infant to a healthcare facility?

- □ No alternative child care
- □ Lack of transportation
- □ Lack of money
- □ The infant did not appear to be seriously ill
- □ Other__________________________

For infant 3: □N/A

28. Date of discharge: _ _/_ _ _/_ _ _ _ (dd/mmm/yyyy) N/A (For home delivery)

29. Time of discharge: _ _/_ _ □AM □PM N/A (For home delivery)

30. Health status at discharge: □Alive □Not alive N/A (For home delivery)

31. Health status at the time of the contact: □Alive □Not alive

32. If not alive, was infant sick at any time since birth?

□No □Yes

33. If yes, was the sick infant brought to healthcare facility for medical care? □No □Yes

34. If no, what is(are) the reason(s) for not bringing the sick infant to a healthcare facility?

- □ No alternative child care
- □ Lack of transportation
- □ Lack of money
- □ The infant did not appear to be seriously ill
- □ Other__________________________

For infant 4: □N/A

35. Date of discharge: _ _/_ _ _/_ _ _ _ (dd/mmm/yyyy) N/A (For home delivery)

36. Time of discharge: _ _/_ _ □AM □PM N/A (For home delivery)

37. Health status at discharge: □Alive □Not alive N/A (For home delivery)

38. Health status at the time of the contact: □Alive □Not alive

39. If not alive, was infant sick at any time since birth?

□No □Yes

40. If yes, was the sick infant brought to healthcare facility for medical care? □No □Yes

41. If no, what is(are) the reason(s) for not bringing the sick infant to a healthcare facility?

- □ No alternative child care
- □ Lack of transportation
- □ Lack of money
- □ The infant did not appear to be seriously ill
- □ Other__________________________

42. Reason(s) for not delivering at the study site
    - ☐ Labor too fast
    - ☐ Family did not allow
    - ☐ No direct transportation to study site
    - ☐ No money for transportation
    - ☐ Did not want to deliver at study site
    - ☐ No childcare available for other child(ren)
    - ☐ Other_____________________________

For site staff:

43. Have you informed the subject about signs and symptoms of potential sepsis in infants?
    - ☐ No
    - ☐ Yes

44. How was the above information obtained?
    - ☐ When the subjects called to provide the information
    - ☐ When the site staff called to obtain information by phone
    - ☐ From the subject during a well child visit
    - ☐ At a home visit
    - ☐ At the baby 90 day follow-up visit
    - ☐ Other____

Survey Questionnaire: Infant 90-day follow-up visit

1. What type of transportation did you use to reach the study site? (Include all applicable)
   - ☐ N/A
   - ☐ Public transportation
   - ☐ Minibus/taxi
   - ☐ Private car
   - ☐ Walk
   - ☐ Ambulance
   - ☐ Other____________________________

2. How much time did it take to reach the study site?
   - ☐ N/A
   - ☐ Less than 15 min
   - ☐ Between 15 and 29 mins
   - ☐ Between 30 and 44 mins
   - ☐ Between 45 and 59 mins
   - ☐ Between 60 and 89 mins
   - ☐ Between 90 and 120 mins
   - ☐ More than 120 mins

3. How much did it cost to reach the study site?
   - ☐ N/A

   ____ (Please also indicate the currency)

4. Do you have access to a telephone to which you can be contacted by the study staff?
   - ☐ No
   - ☐ Yes, provide all numbers here.

     Home phone___________________________________
     Personal mobile phone___________________________
     Other____
     (Phone number(s) is(are) only for the register of the site. They will not be included in the eCRF)

5. Do you have other child(ren) under your care at this time?
   - ☐ No
   - ☐ Yes. Is anyone else looking after them while you are in hospital?
     - ☐ No
     - ☐ Yes
     - ☐ N/A

For infant 1:

6. Health status of the infant at this time: □Alive □Not alive
7. Did you bring your infant to well child / immunization visits: □No □Yes
   - □ If yes, number of visits:______________
8. Was this infant sick at any time since birth? □No □Yes
9. If yes, was the sick infant brought to a healthcare facility for medical care? □No □Yes
10. If yes, to which healthcare facility did you bring your sick infant for medical care?
    - □ To the study site
    - □ To the infant's hospital/clinic for immunization
    - □ Other

    Please write the name of the other hospital/clinic_______________________
11. If no, what is (are) the reason(s) for not bringing the infant to a healthcare facility?
    - □ No alternative child care
    - □ Lack of transportation
    - □ Lack of money
    - □ The infant did not appear to be seriously ill
    - □ Other__________________________

For infant 2 N/A□

12. Health status of the infant at this time: □Alive □Not alive
13. Did you bring your infant to well child / immunization visits: □No □Yes
    - □ If yes, number of visits:______________
14. Was this infant sick at any time since birth? □No □Yes
15. If yes, was the sick infant brought to a healthcare facility for medical care? □No □Yes
16. If yes, to which healthcare facility did you bring your sick infant for medical care?
    - □ To the study site
    - □ To the infant's hospital/clinic for immunization
    - □ Other

    Please write the name of the other hospital/clinic_______________________
17. If no, what is (are) the reason(s) for not bringing the infant to a healthcare facility?
    - □ No alternative child care
    - □ Lack of transportation
    - □ Lack of money
    - □ The infant did not appear to be seriously ill
    - □ Other__________________________

For infant 3 N/A□

18. Health status of the infant at this time: □Alive □Not alive
19. Did you bring your infant to well child / immunization visits: □No □Yes
    - □ If yes, number of visits:______________

20. Was this infant sick at any time since birth? □No □Yes
21. If yes, was the sick infant brought to a healthcare facility for medical care? □No □Yes
22. If yes, to which healthcare facility did you bring your sick infant for medical care?
    - □ To the study site
    - □ To the infant's hospital/clinic for immunization
    - □ Other

    Please write the name of the other hospital/clinic______________________
23. If no, what is (are) the reason(s) for not bringing the infant to a healthcare facility?
    - □ No alternative child care
    - □ Lack of transportation
    - □ Lack of money
    - □ The infant did not appear to be seriously ill
    - □ Other__________________________

For infant 4 N/A□

24. Health status of the infant at this time: □Alive □Not alive
25. Did you bring your infant to well child / immunization visits: □No □Yes
    - □ If yes, number of visits:_____________
26. Was this infant sick at any time since birth? □No □Yes
27. If yes, was the sick infant brought to a healthcare facility for medical care? □No □Yes
28. If yes, to which healthcare facility did you bring your sick infant for medical care?
    - □ To the study site
    - □ To the infant's hospital/clinic for immunization
    - □ Other

    Please write the name of the other hospital/clinic______________________
29. If no, what is (are) the reason(s) for not bringing the infant to a healthcare facility?
    - □ No alternative child care
    - □ Lack of transportation
    - □ Lack of money
    - □ The infant did not appear to be seriously ill
    - □ Other__________________________

For site staff:

30. Did you obtain the information about delivery at visit 2? □No □Yes

31. The above information was obtained:
    - □ At the visit at study site, date of visit of the subject: _ _/_ _ _/_ _ _ _ (dd/mmm/yyyy)
    - □ When the site staff called to obtain information by phone
    - □ From the subject during a well child visit

- ☐ At a home visit
- ☐ Other____
