# Supplementary material for: Factors influencing access of pregnant women and their infants to their local healthcare system: a prospective, multi-centre, observational study
Source: BMC Pregnancy Childbirth. 2018 Jan 15;18:29. doi: 10.1186/s12884-017-1655-3 (PMC5769295; doi:10.1186/s12884-017-1655-3)
Supplement: Supplementary file 2 — Supplementary Methods. Formula and details on odds ratios calculation. (PDF 85 kb) [file 12884_2017_1655_MOESM2_ESM.pdf]

Additional file 2

Factors influencing access of pregnant women and their infants to their local healthcare system: a prospective, multi-centre, observational study

Shabir A. Madhi et al.

Supplementary Methods

Odds ratios were calculated by fitting the following fixed effect logistic model:

$$logit(y_{ij}) = \beta_0 + \sum_{k=1}^{n} \beta_{kl} x_{ijk} \mathrm{I}_{[x_{ijk}=l]} + \sum_{k=n+1}^{n+m} \gamma_k x_{ijk}$$

Where the logit(y_{ij}) is the P(Y=1)/[1-P(Y=1)], β_0 is the intercept parameter, β_{kl} is the vector of regression parameter for the l-th level of the categorical variable x_{ijk}, k=1, 2, …, n , l=1, 2, …, L_{k-1} where L_k is the reference group; and, γ_k is the slope parameter for the continuous variable x_{ijk}, k=n+1, n+2, …, n+m. Note that $I_{[x_{ijk}=l]}$ is the indicator that the categorical variable x_{ijk} is the value of the l-th level.
